# Supplementary material for: Acoustic analogues of three-dimensional topological insulators
Source: Nat Commun. 2020 May 8;11:2318. doi: 10.1038/s41467-020-16131-w (PMC7211004; doi:10.1038/s41467-020-16131-w)
Supplement: Supplementary file 1 — Supplementary Information [file 41467_2020_16131_MOESM1_ESM.pdf]

# Supplementary Information

## Acoustic analogues of three-dimensional topological insulators

He et al.

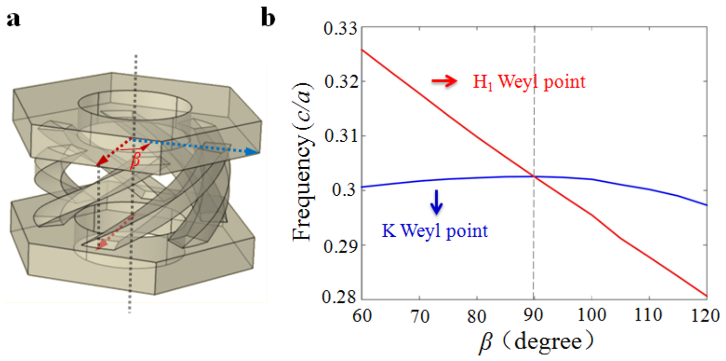


**Supplementary Figure 1 Iso-frequency Weyl points based on the spiral angle. a** Single-layer unit cell of the chiral structure, where $\beta$ represents the spiral angle of the air channels. **b** The frequencies of Weyl points at K (blue line) and $H_1$ (red line) points with various $\beta$ from 60 ° to 120 °. They are in the same frequency when $\beta$=90 °. Other parameters are the same as those of Fig. 1b in the main text.

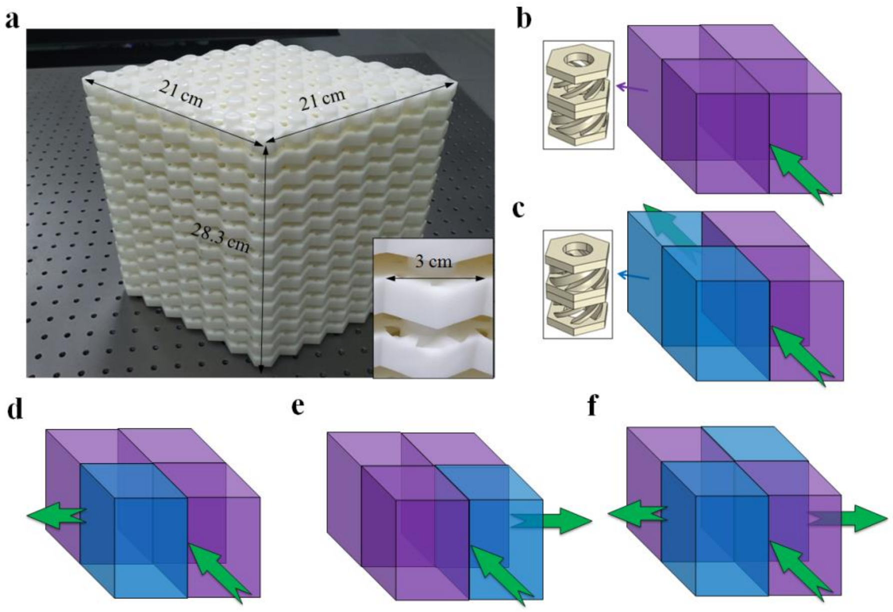


**Supplementary Figure 2 Guide of sound by building blocks. a** One of four uniform rhomb–prisms (7×7×7 unit cells). The inset shows the lateral view. **b-c** Experimental configurations to measure the bulk and surface transmission spectra of Fig. 2d in the main text. The purple and blue regions represent oppositely placing of the rhomb–prisms (respective to the *z* axis). **d-f** Experimental configurations to measure a 60° bend, a 120° bend, and splitter transmission spectra of Fig. 2e in the main text.

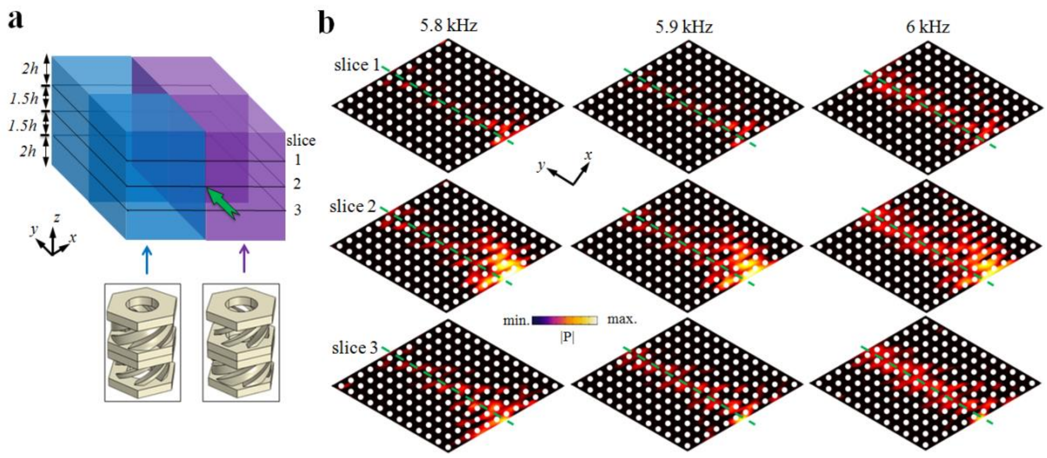


**Supplementary Figure 3 Experimentally measured acoustic fields for straight configuration. a** Schematic of configuration to measure acoustic fields for three slices with *2h*, *3.5h* and *5h*, respectively. **b** Measured acoustic fields for the three slices at various frequencies 5.8, 5.9 and 6 kHz, respectively. The colour scale represents the absolute value of acoustic pressure (P).

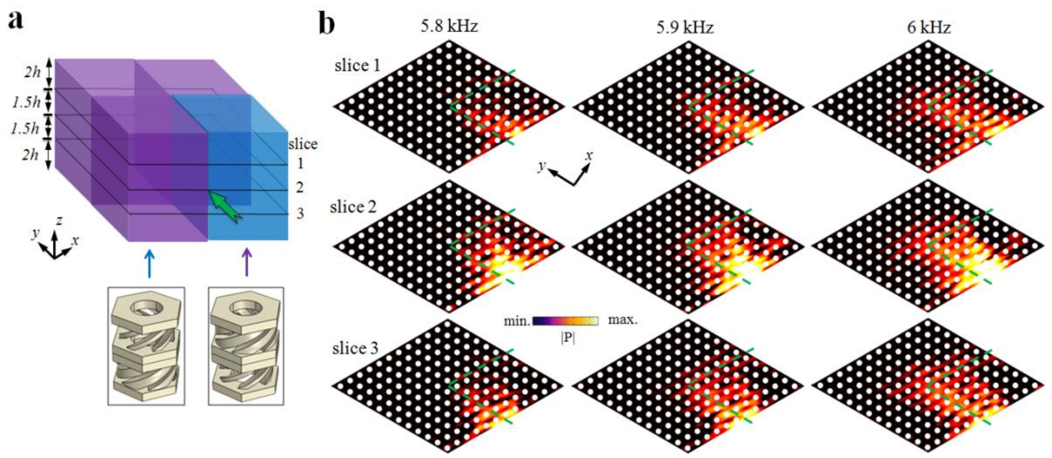


**Supplementary Figure 4 Experimentally measured acoustic fields for a 120 ° bend configuration.** **a** Schematic of configuration. **b** Measured acoustic fields. The colour scale represents the absolute value of acoustic pressure.

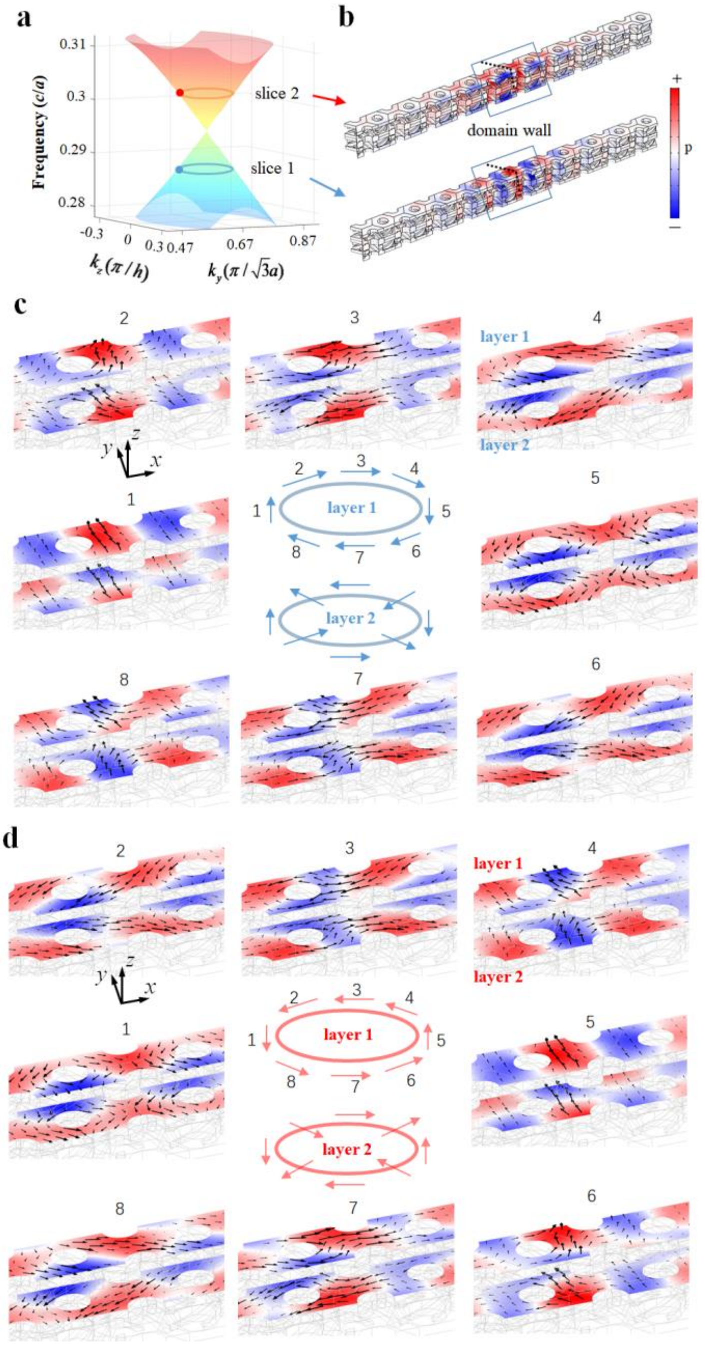


**Supplementary Figure 5 Acoustic pseudospin textures.** **a** Surface Dirac cone in the $k_{yz}$ plane (Fig. 2c in the main text). **b** Bloch field distributions for two slices (red and blue points in panel **a**). **c** Acoustic pseudospin textures of the slice 1, which are composed of in-phase (e.g. points 1 and 5) and out-of-phase (e.g. points 3 and 7) vibrations in two different layers[1]. **d** Acoustic pseudospin textures of the slice 2. Here, the black arrows represent acoustic intensity vectors in the *xy* plane. The colour scale represents the acoustic pressure.

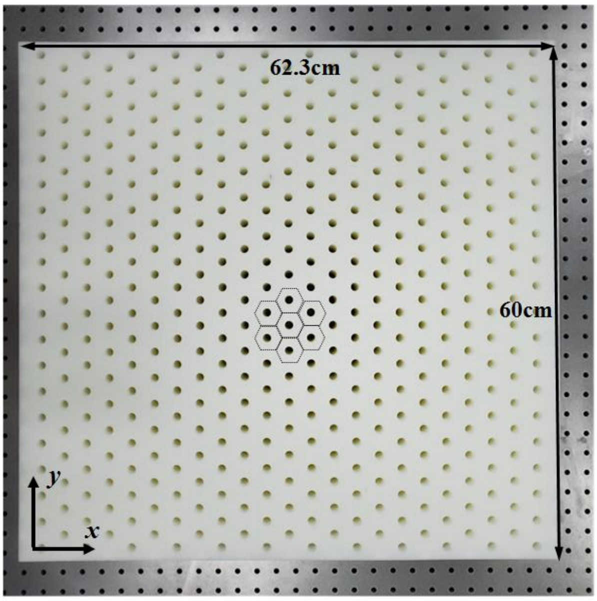


**Supplementary Figure 6 Experimental sample to measure 2D surface Dirac cone.** To map the surface Dirac cone of Fig. 3c in the main text, our sample is fabricated with 24×20 unit cells in the *xy* plane. Thus, the resolution of measured Bloch momentum is better than $0.1\times 4\pi/3\sqrt{3}a$ (0.1×4π/9 cm$^{-1}$**).** 2D Fast Fourier transform of acoustic pressure fields with zero padding method is used to obtain Fig. 3c in the main text. In fabrication, we hollow the holes to save materials.

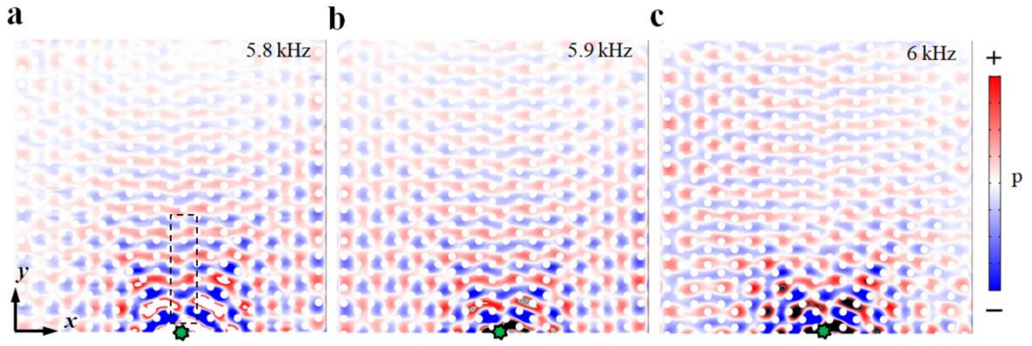


**Supplementary Figure 7 Experimentally measured acoustic fields for the top surface (the sample shown in Supplementary Figure 6).** Measured acoustic fields at various frequencies **a** 5.8 kHz, **b** 5.9 kHz, **c** 6 kHz**.** The colour scale represents the acoustic pressure.

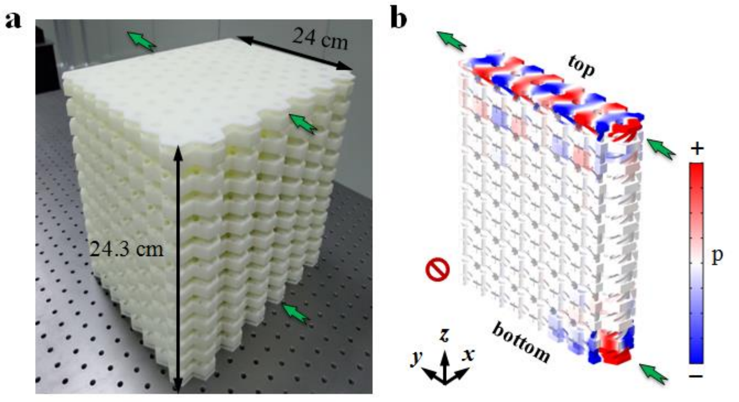


**Supplementary Figure 8 Experimental sample to measure robust sound transport in the *xy* plane.** **a** Experimental sample with hard boundaries (both the top and bottom surfaces are covered by plastic boards). We measure the sound transmission spectra with acoustic sources excited at the top and bottom. The results are shown in Fig. 3d of the main text. **b** A slice of simulated acoustic pressure distribution with acoustic source excited at both the top and bottom surfaces at a frequency of 5.8 kHz (the same as Fig. 3e in the main text), indicating the sound only propagates at the top surface in this case. Here, *yz* plane are set to be continuous condition in simulation. The colour scale represents the acoustic pressure.

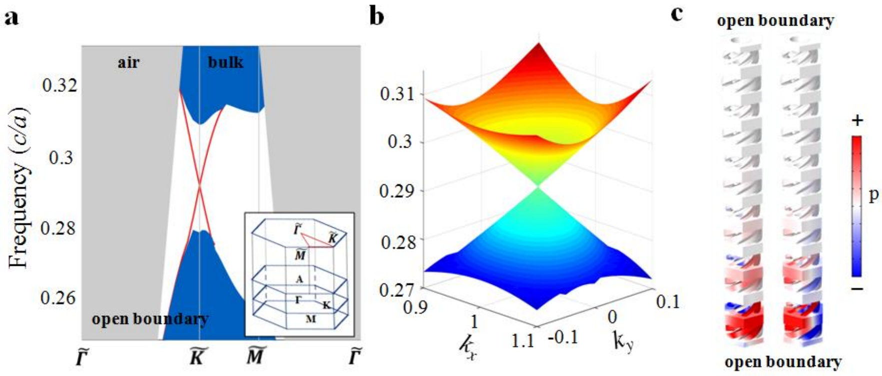


**Supplementary Figure 9 Open boundary case. a** Projection of bandstructures in the *xy* plane with an open boundary condition, where surface Dirac cones appear at the bottom surface. The shadow region (gray colour) indicates the air cone for sound. **b** 3D view of the surface Dirac cone. Here, the $k_x$ and $k_y$ have units of 4π/9 $cm^{-1}$. **c** Bloch field distributions for the open boundary case, where the colour represents the acoustic pressure.

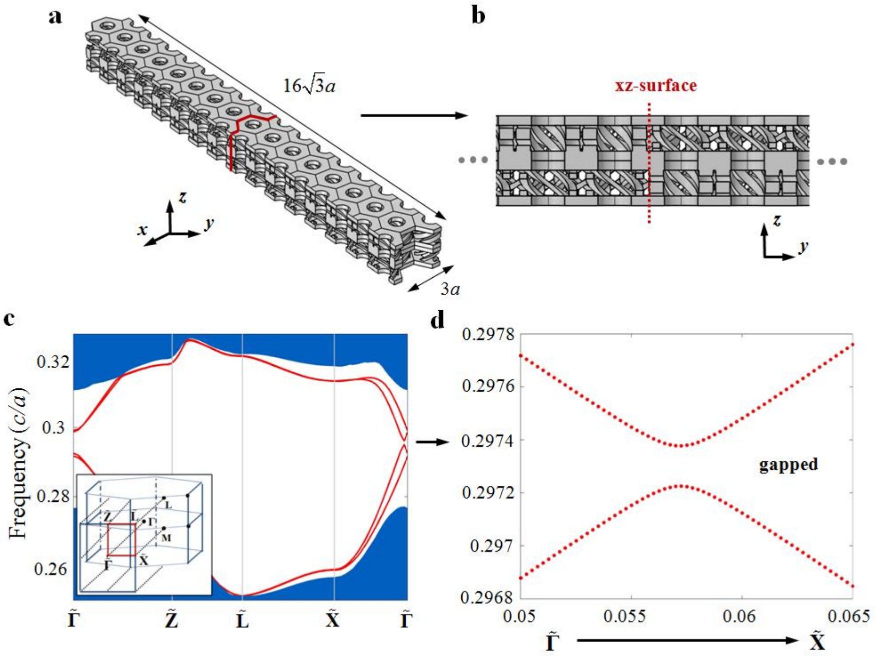


**Supplementary Figure 10 Topological trivial surface in the *xz*-domain wall. a-b** Supercell configuration and its zoom-in lateral view of the *xz*-domain wall (armchair interface). **c** Projected bandstructures in the $k_{xz}$ domain wall, where the red lines represent surface states. The inset shows the highly symmetric directions of surface Brillouin zone. **d** Zoom-in projected bandstructures to show the gapped surface states.

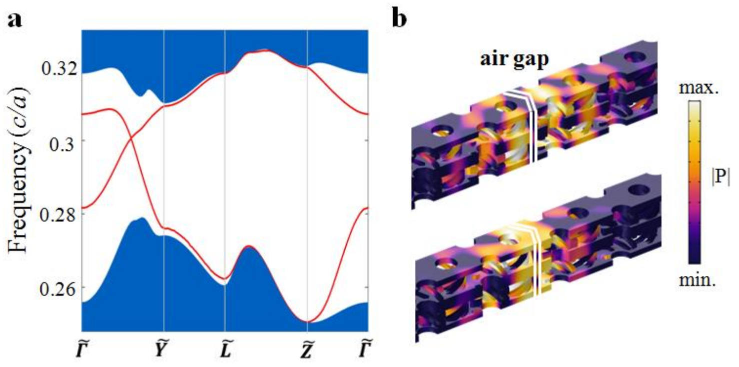


**Supplementary Figure 11 Robustness in the presence of an air gap.** In Fig. 2 of the main text, different configurations are assembled by four rhomb–prism segments. An air gap may exist when putting them together, however, the topological behaviour associated with the surface Dirac cones remain unchanged. **a** Projected bandstructures in the *yz* domain wall in the case of $a/2\sqrt{3}$ thickness air gap are introduced (corresponding to 0.5 cm in experiments). **b** Bloch field distributions at the frequency of surface Dirac point, where the colour scale represents the absolute value of acoustic pressure.

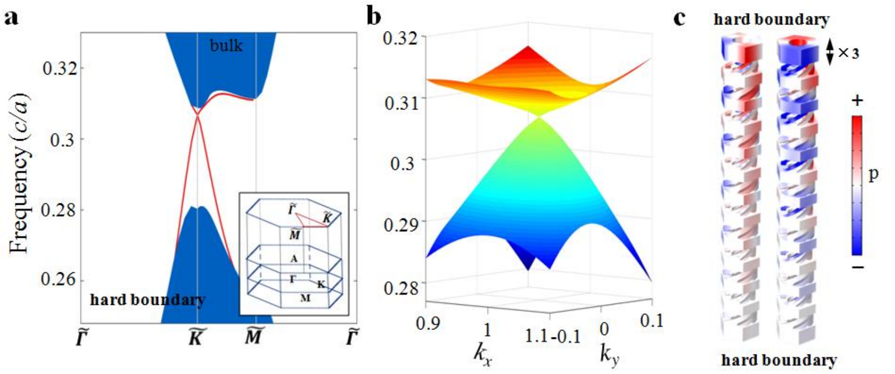


**Supplementary Figure 12 Robustness against boundary thickness. a** Projection of bandstructures in the *xy* surface with a hard boundary condition. Here, we increase the thickness of the top and bottom air layers three times thicker than those in Fig. 3 of the main text. The gapless surface Dirac cones still exist at the top surface except with a higher surface Dirac crossing frequency. **b** 3D view of surface Dirac cone. Here, $k_x$ and $k_y$ are have units of 4π/9 cm$^{-1}$. **c** the Bloch field distributions, where the colour represents acoustic pressure.

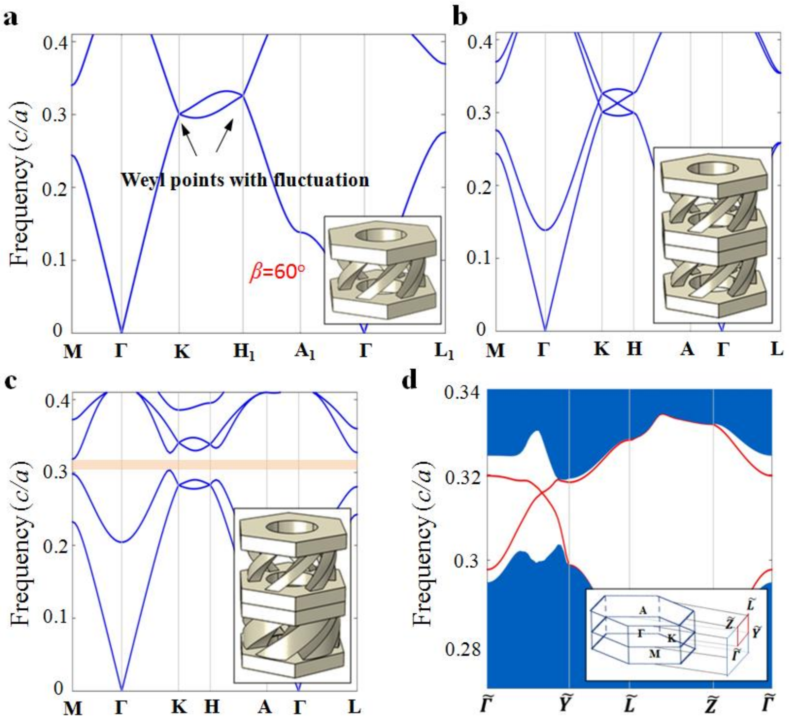


**Supplementary Figure 13 Robustness against the spiral angle.** Here, we change the spiral angle $\beta$ from 90° to 60°. Other parameters are the same as those of Fig. 1b in the main text. Bulk bandstructures for **a** single-layer case, **b** bilayer case, **c** symmetry-breaking case corresponding to Figs. 1b-d in the main text. The insets show the unit cells. **d** Surface Dirac cones can also be found in the *yz* domain wall.

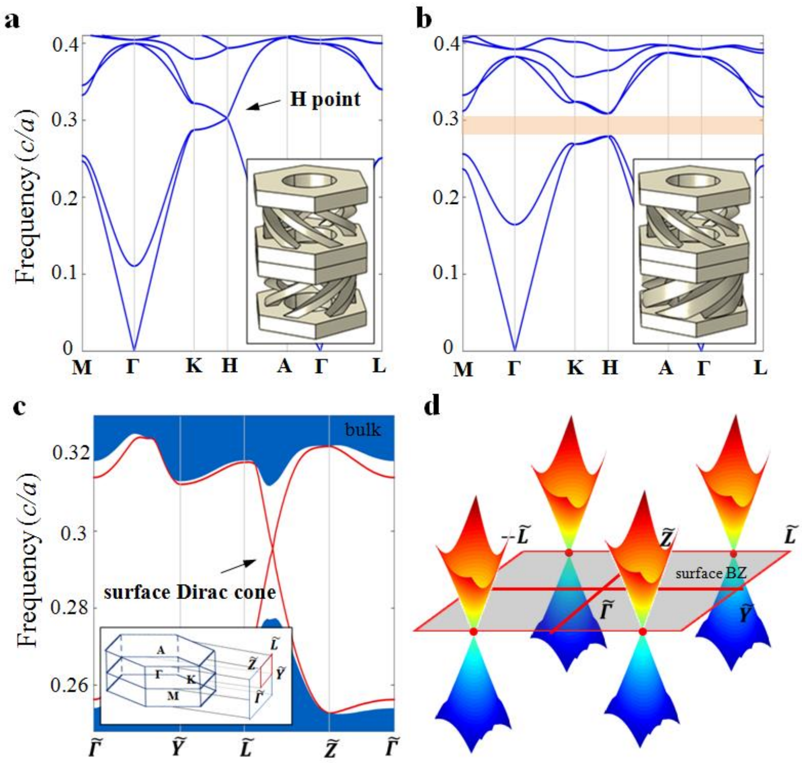


**Supplementary Figure 14 Glide symmetry case to 3D acoustic TI.** In the main text, we use doubling strategy to construct double Dirac cone at K pint as shown in Figs. 1c and 1f. We can also fold the Weyl points to H point by introducing glide symmetry of the bilayer unit cell. **a** Bulk bandstructures for glide-symmetric bilayer structure. **b** Topological bandgap with breaking glide symmetry. **c** Projection bandstructures in the *yz*-domain wall. **d** Schematic of surface Dirac cones located at the boundaries of surface Brillouin zone.

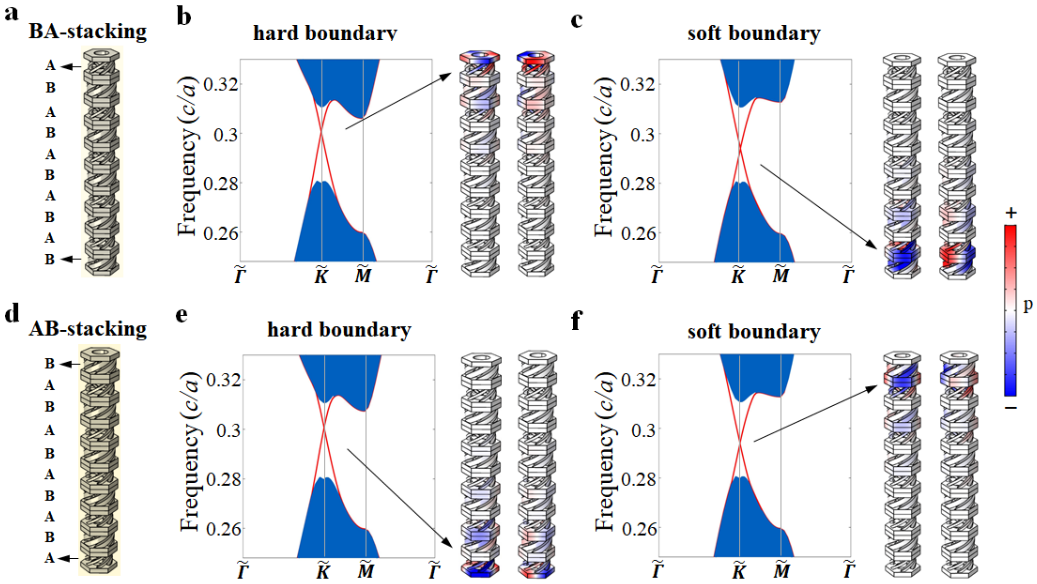


**Supplementary Figure 15 Intact boundary cases with hard and soft boundaries. a** BA-stacking supercell configuration, where A represents the small spiral channel layer while B represents the large spiral channel layer. **b** Projected bandstructures and Bloch field distributions with hard boundaries, where the surface states located at the top (A layer). **c** Soft boundary case, where the surface states located at the bottom (B layer). **d** AB-stacking supercell configuration. **e** Hard boundary case, where the surface states located at the bottom (A layer). **f** Soft boundary case, where the surface states located at the top (B layer). The colour scale represents the acoustic pressure. This situation is similar to 3D topological photonic case with perfect-electric-conductor boundary or perfect-magnetic-conductor boundary[2].

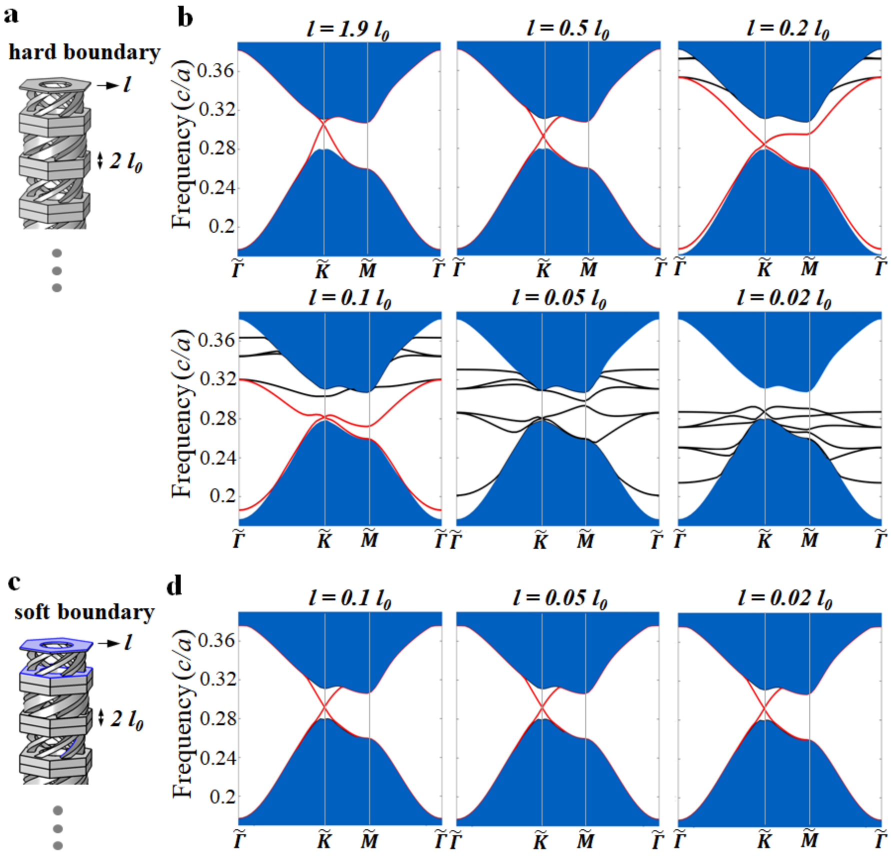


**Supplementary Figure 16 Truncated surface cases with the small spiral channel layer. a** Supercell configuration with hard boundaries. **b** Projected bandstructures with various air layer thickness ($l$): $1.9l_0$, $0.5l_0$, $0.2l_0$, $0.1l_0$, $0.05l_0$, and $0.02l_0$. With decreasing the thickness, the trivial surface states (black lines) will hybridize the nontrivial ones (red lines) to break the gapless property. **c** Supercell configuration with soft boundaries (blue planes). **d** Projected bandstructures with gapless surface states.

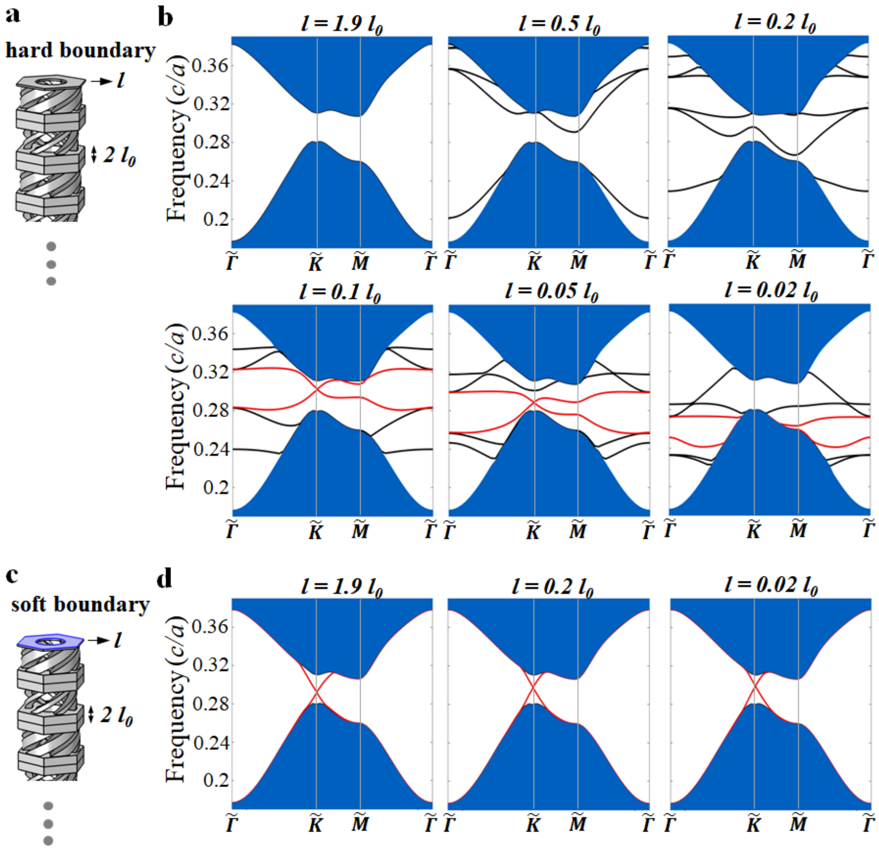


**Supplementary Figure 17 Truncated surface cases with the large spiral channel layer.** **a** Supercell configuration with hard boundaries. **b** Projected bandstructures with various thickness: $1.9l_0$, $0.5l_0$, $0.2l_0$, $0.1l_0$, $0.05l_0$, and $0.02l_0$. With decreasing the thickness, the nontrivial surface states (red lines) may appear, however, the trivial ones (black lines) also exist. **c** Supercell configuration with soft boundary (blue plane). **d** Projected bandstructures with gapless surface states.

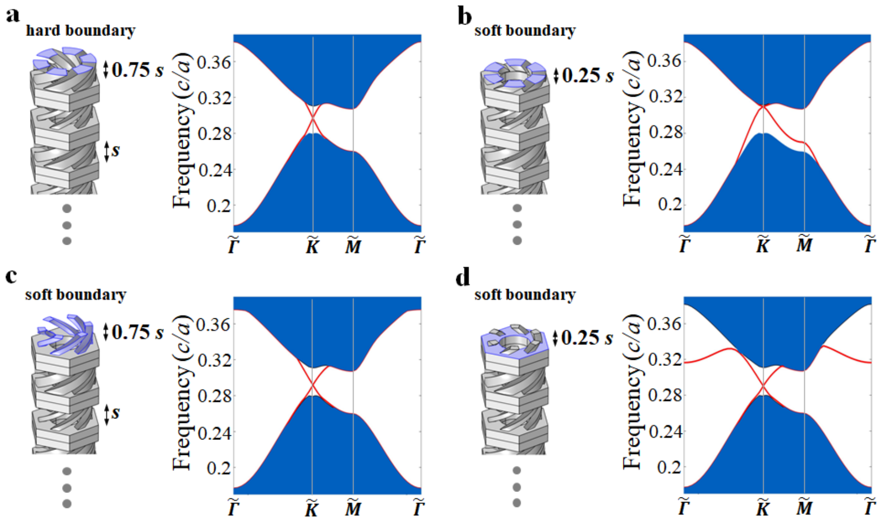


**Supplementary Figure 18 Truncated spiral channel cases. a** Supercell configuration and the projected bandstructures with truncated large spiral with hard boundary (0.75*s*). **b** Soft boundary case (0.25*s*). **c** Truncated small spiral case with hard boundary (0.75*s*). **d** Soft boundary case (0.25*s*).

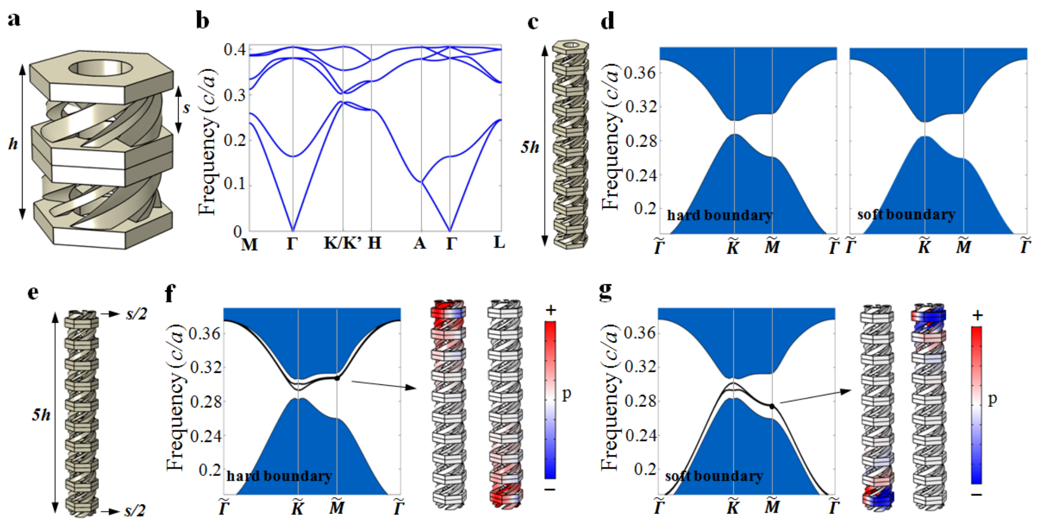


**Supplementary Figure 19 Alternate chiral channel case. a** Alternate chiral channels used in the same layer to break the degeneracy. **b** Bulk bandstructures with a full gap. **c** Supercell configuration. **d** Projected bandstructures in the $k_{xy}$ plane with hard and soft boundary conditions, where no surface states can be observed. **e** Supercell configuration with truncated surfaces. **f** Projected bandstructures and Bloch field distributions with hard boundaries. **g** Soft boundary condition. Only the trivial surface states can be observed. The colour scale represents the acoustic pressure.

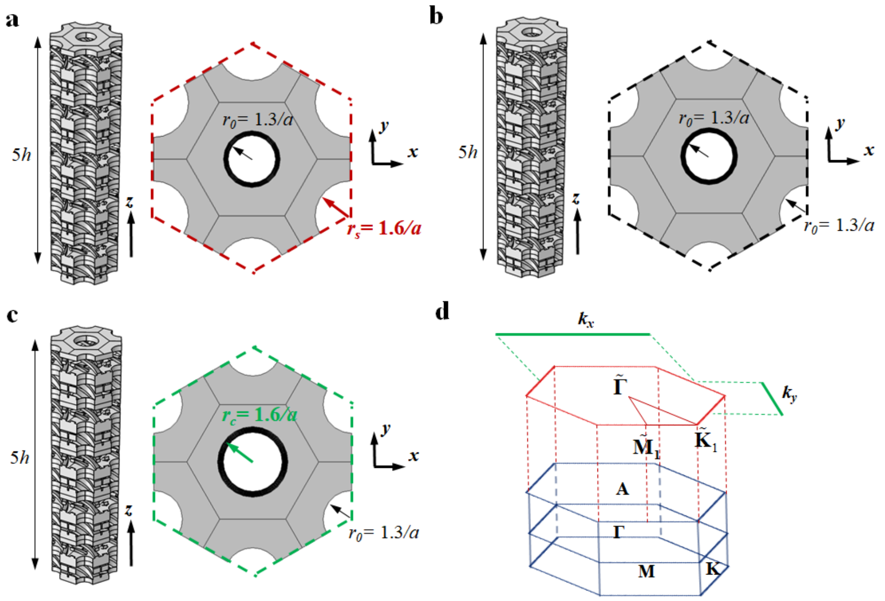


**Supplementary Figure 20 Supercell configurations to obtain 2nd-order topological bandgap. a-c** Supercell configurations and their top-down views used to calculate the bandstructures of Figs. 4a-c in the main text. **d** The 3D bulk Brillouin zone projected to the 2D *xy* surface (red lines) and 1D hinge Brillouin zones (green lines).

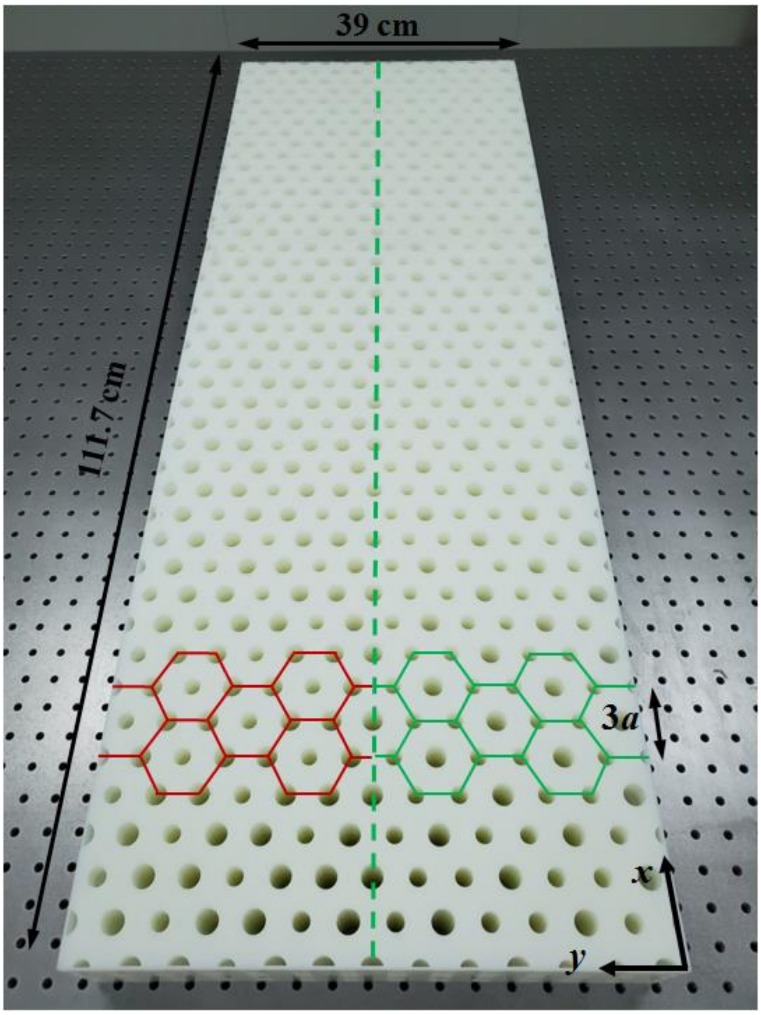


**Supplementary Figure 21 Experimental sample to measure 1D hinge Dirac dispersion.** To map 1D hinge Dirac dispersion (Fig. 4e in the main text), our sample is fabricated longer than $20\times 3a$ along the $x$ axis. Thus, the resolution is better than $0.1\times\pi/3a$ . In fabrication, we hollow the holes to save materials.

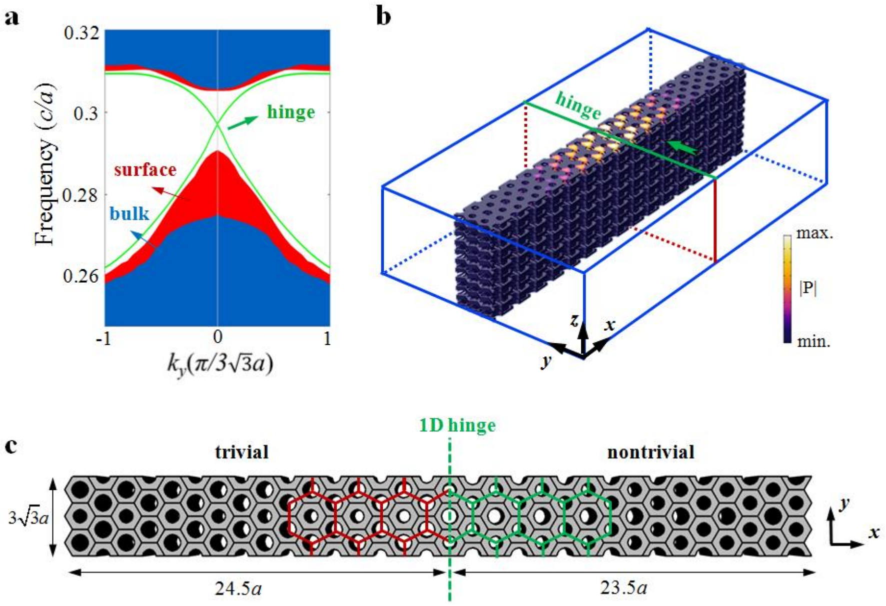


**Supplementary Figure 22 1D gapless hinge states along the $k_y$ direction. a** The projection of 3D bandstructures in the 1D $k_y$ direction. **b** Simulated Bloch field distributions at $k_y$=0 point, where the colour scale represents the absolute value of acoustic pressure. **c** Top-down view of supercell configuration to calculate projected bandstructures, where the dashed green line represents the 1D hinge along the *y* axis.

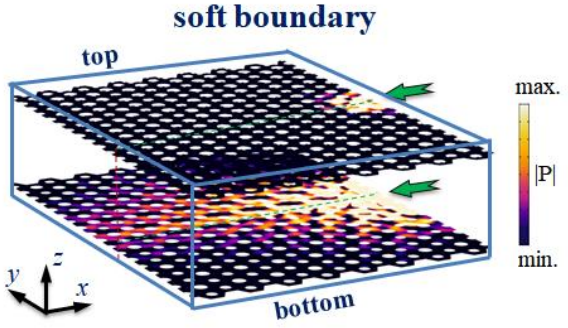


**Supplementary Figure 23 Soft boundary condition for hinge state compared to Fig. 4g in the main text.** Simulated acoustic pressure distributions for the top and bottom hinges at a frequency of 5.81 kHz, where the colour scale represents the absolute value of acoustic pressure.

**Supplementary Methods**

In this section, we construct a tight-binding lattice model for the 3D bilayer chiral honeycomb acoustic crystal to obtain the effective Hamiltonian in the vicinity of K point. As shown in Fig. 1b of the main text, the single-layer chiral acoustic structure can be viewed as a stacked honeycomb lattice, and each honeycomb lattice contains two sub-lattices. If we omit sub-lattice onsite energy difference for simplicity, the Hamiltonian of this system can be described as a two-level effective Hamiltonian of the acoustic Weyl system[3]:

$$H_{sl} = \begin{bmatrix} t_c f(k_z) & t_n \beta \\ (t_n \beta)^* & t_c f(-k_z) \end{bmatrix}, \tag{1}$$

where $\beta = e^{-ik_x a} + 2cos(\sqrt{3}k_y a/2)e^{-ik_x a}$ and $f(k_z) = 2\left[\cos\left(\frac{5k_x a}{4} + \frac{\sqrt{3}k_y a}{2} - \frac{k_z h}{2}\right) + \cos\left(k_x a - \frac{\sqrt{3}k_y a}{2} + \frac{k_z h}{2}\right) + \cos\left(\frac{k_x a}{4} + \frac{3\sqrt{3}k_y a}{4} + \frac{k_z h}{2}\right)\right]$.

In Supplementary Equation 1, $t_n$ denotes the intralayer hopping strengths between nearest sub-lattices, and $t_c$ denotes the interlayer hopping terms. Then, we can expand the Hamiltonian around K point (also for $H_1$ point) up to linear $k$:

$$H_{sl} = (\varepsilon + \chi k_x + \lambda k_y)\sigma_0 + v_f(k_y \sigma_x - k_x \sigma_y) + v_d k_z \sigma_z, \tag{2}$$

where $\sigma_i$ $(i=x,y,z)$ are Pauli matrices, $\sigma_0$ is the identity matrix, $v_f$ and $v_d$ are the $xy$-plane and out-of-plane acoustic group velocities, $\varepsilon$ represents an overall shift of the bands, $\chi$ and $\lambda$ represent the tilt from the $x$ axis and $y$ axis. Thus, we have recovered the Weyl equation, corresponding to Fig. 1e of the main text.

Based on this Weyl equation, we can construct the double Dirac cone via Brillouin zone folding mechanism as shown in Fig. 1c and Fig. 1f of the main text. In particular, we double single-layer chiral structure to form a bilayer unit cell. Thus, the Hilbert space of such bilayer model can be described as the direct sum of Hilbert spaces of two single-layer models. Considering the additional interlayer coupling of adjacent crystals and intralayer hopping, the final Hamiltonian can be written as:

$$H=\begin{bmatrix}(t_{c1}-t_{c2})\cos(k_zh) & 0 & \frac{(t_{c1}+t_{c2})f(k_z)}{2} & t_n\beta \\ 0 & (t_{c1}-t_{c2})\cos(k_zh) & (t_n\beta)^* & \frac{(t_{c1}+t_{c2})f(-k_z)}{2} \\ \frac{(t_{c1}+t_{c2})f(k_z)}{2} & t_n\beta & (t_{c2}-t_{c1})\cos(k_zh) & 0 \\ (t_n\beta)^* & \frac{(t_{c1}+t_{c2})f(-k_z)}{2} & 0 & (t_{c2}-t_{c1})\cos(k_zh)\end{bmatrix}, \quad (3)$$

where $t_{c1}$ ($t_{c2}$) represent the interlayer hopping strength of each lower (upper) single-layer chiral structure. The off-diagonal terms denote the intralayer coupling and the diagonal terms denote interlayer coupling. By defining $m=t_{c1}-t_{c2}$ (the effective mass term), we can get the 3D massive Dirac Hamiltonian for bulk bands:

$$H_{bulk}=\tau_x v_f(k_y\sigma_x-k_x\sigma_y)+v_dk_z\tau_x\sigma_z+m\tau_z\sigma_0. \quad (4)$$

Here, we omit ε, χ, λ for simplicity since they only represent the overall shift or tilt of the bands. $\tau_i$ $(i=x,y,z)$ are Pauli matrices acting on acoustic pseudospin space. The Hamiltonian depicted in In Supplementary Equation 4 is similar to Bernevig-Hughes-Zhang model of 3D TI in electronic systems[4,5]. When $t_{c1}-t_{c2}=0$, i.e., the same spiral channels between the lower and upper layers, Supplementary Equation 4 becomes a 3D massless Dirac Hamiltonian with the 3D Double Dirac points at K and K' points (Fig. 1c and Fig. 1f of the main text). On the contrary, if we break the $h/2$ translational symmetry by enlarging the spiral channels of the lower layer (Fig. 1d and Fig. 1g of the main text), the Dirac Hamiltonian becomes massive, and a topological bandgap appears.

To obtain the Dirac-like surface sates in the system, we consider a hard boundary condition with an interface located at $z$=0 plane. With the hard boundary condition $\partial\psi/\partial z|_{z=0}=0$ and $\psi(z\to\infty)=0$, we can get the effective Hamiltonian for the surface states[6]:

$$H_{surface}=v_f(k_y\sigma_x-k_x\sigma_y). \quad (5)$$

Supplementary Equation 5 represents a Dirac-like surface dispersion within the bulk bandgap, corresponding to Fig. 3 of the main text. It should be noticed that Supplementary Equation 3 can also

be expanded around the K' valley and we can obtain the same Dirac cone-like surface state near the K' point.

Above discussion deals with the 2D gapless surface states within the 1st-order topological bandgap in a 3D acoustic crystal. Notably, the top surface along the *z* direction preserves the $C_{6v}$ symmetry that can further be utilized to realize the 2nd-order TIs with 1D hinge states. Similarly, the first step is to construct the 4-fold Dirac degeneracy[7]. Here, we utilize the above two surface Dirac cones at K and K' points to construct double surface Dirac cone that is 4-fold degeneracy for topological surface states. Different from the bulk bands folding along the *z* axis in Fig. 1b and Fig. 1c of the main text, here we fold the surface bands in the $xy$ plane. We select a triple size honeycomb lattice as a new unit cell, which is three times larger than the original bilayer unit cell (Fig. 1c of the main text). Consequently, Brillouin zone is reduced to one third, and the two surface Dirac cones at K and K' points are folded into the center of the new surface Brillouin zone[8]. Then, we selectively change the radii of the center holes or the side holes to break the surface Dirac degeneracy. The surface dispersion will undergo a process from trivial to close, then to topologically nontrivial bandgap. The edges of 2D surfaces are 1D hinges. Considering a hinge ($z$=0 and $y$=0) constructed with the topological trivial and nontrivial surface at each side, we can get effective Hamiltonian for hinge states:

$$H_{hinge} = -v_f k_x \sigma_z. \quad (6)$$

Thus, we obtain both 3D 1st-order acoustic TI with 2D gapless Dirac-like surface states (Supplementary Equation 5) and 3D 2nd-order acoustic TI with 1D gapless Dirac-like hinge states (Supplementary Equation 6), which are protected by the topological nature of 3D bulk.

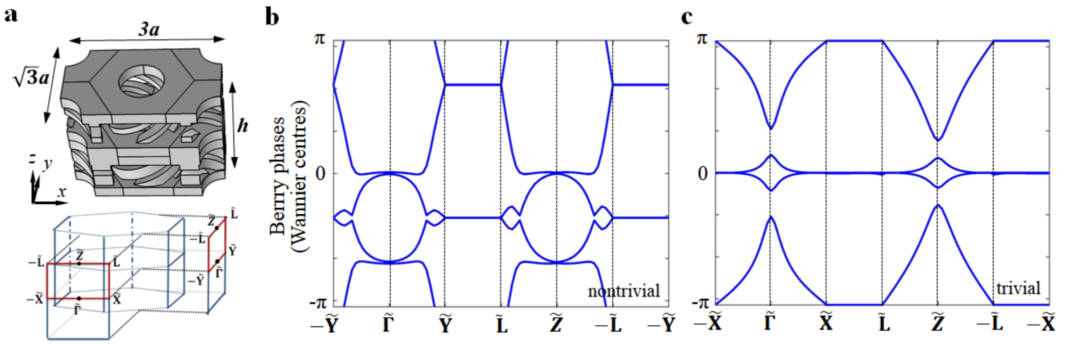


**Supplementary Figure 24 Hybrid Wannier centers. a** Schematic of an orthogonal unit cell. **b** Eigenvalues of a Wilson loop of four bulk bands below the bandgap along the *x* direction corresponding to Fig. 2 in the main text. **c** Eigenvalues of a Wilson loop along the *y* direction corresponding to Supplementary Figure 10. Here, to match the surface Brillouin zones of Fig 2b in the main text and Supplementary Figure 10, we use an orthogonal unit cell to carry out our calculations. The orthogonal unit cell is two times larger than the primary unit cell (Fig. 1d in the main text). Therefore, the first two bulk bands below the bandgap (Fig. 1g in the main text) are folded into four bands; however, their topological properties are the same.

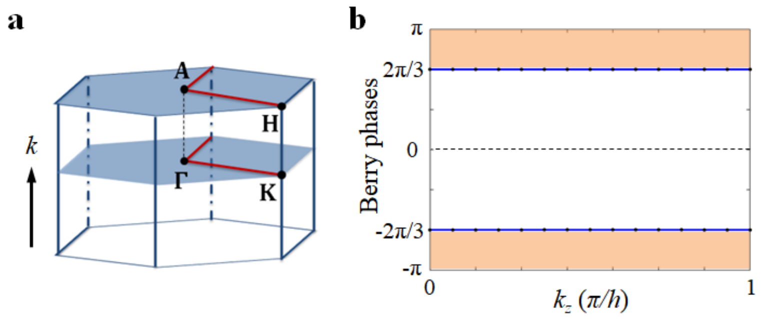


**Supplementary Figure 25 Bent Wilson loop[9]. a** Schematic of a bent loop in the 3D Brillouin zone of hexagonal lattice. **b** Berry-phase spectrum of a bent Wilson loop along the *z* direction.

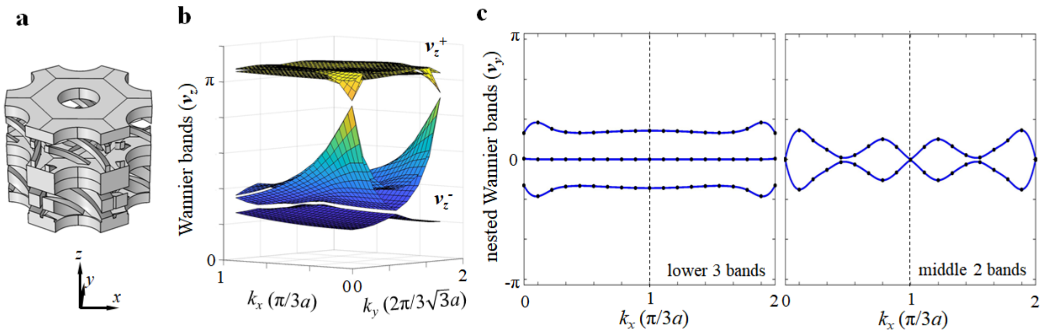


**Supplementary Figure 26 Nested Wilson loop for trivial case. a** Schematic of an unit cell Fig. 4a in the main text). **b** Numerically calculated 2D Wannier bands of a Wilson loop along the *z* direction. **c** Numerically calculated 1D Wannier bands of a nested Wilson loop for lower three bands ($\boldsymbol{v_z}^-$ in panel **b**) and for middle two bands along the *y* direction. The 1D nested Wannier bands show trivial character for hinge state with Wannier center located at $v_y$=0.

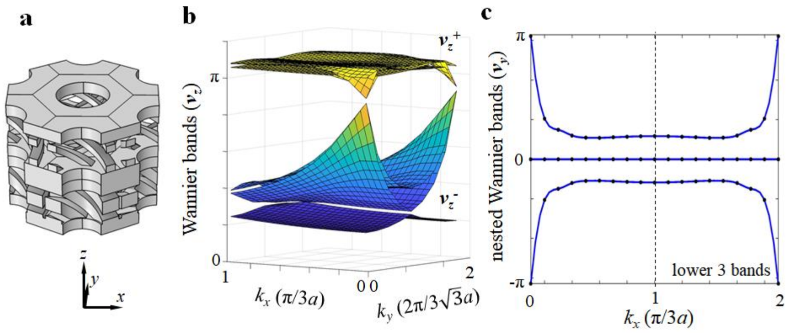


**Supplementary Figure 27 Nested Wilson loop for nontrivial case. a** Schematic of an unit cell (Fig. 4c in the main text). **b** Numerically calculated 2D Wannier bands of a Wilson loop along the *z* direction. **c** Numerically calculated 1D Wannier bands of a nested Wilson loop for lower three bands along the *y* direction. Here, two separated Wannier sectors (three bands in each one) can be obtained. We calculate the nested Wannier loop for lower three bands along the *y* direction. Besides $v_y$=0, the Wannier centers can also be found at $v_y$=±π. It means that such hinge state is a phononic obstructed atomic limit case, which is different from trivial cases whose Wannier centers always locate at the origin. On the other hand, such 1D nested Wannier bands in our 3D acoustic system are similar to 1D Wannier bands of 2D fragile acoustic TI after considering a set of three bulk bands the below bandgap, which can be treated as a kind of acoustic analogue of 3D 2$^{nd}$-order TI[10-13].

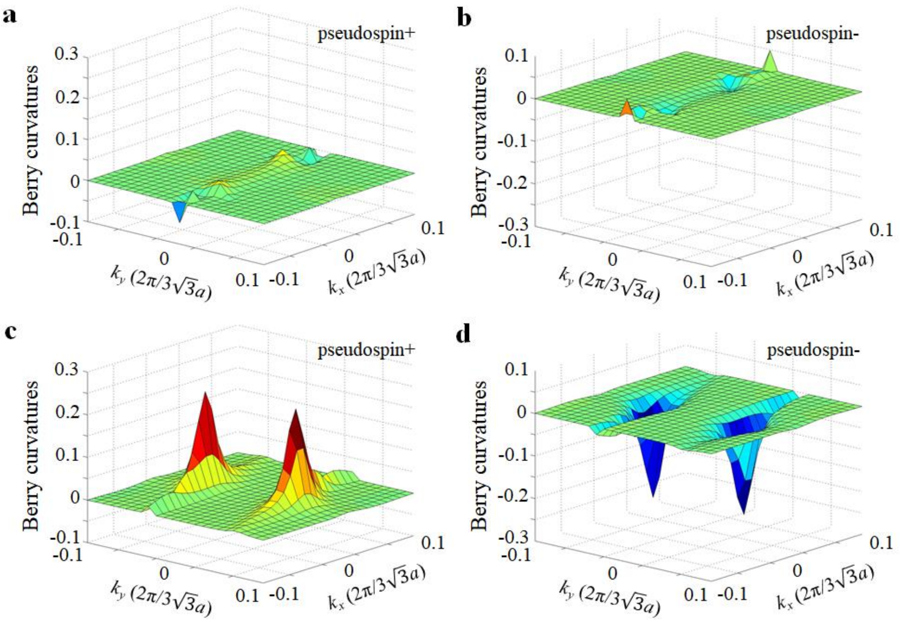


**Supplementary Figure 28 Berry curvatures of the surface states near the center of surface Brillouin zone. a-b** Trivial case corresponding to Fig. 4a in the main text. **c-d** Nontrivial case corresponding to Fig. 4c in the main text.

**Supplementary References**


1. Liu, Y., Xu, Y. & Duan, W. Three-dimensional topological states of phonons with tunable pseudospin physics. *Research* **2019**, 5173580 (2019).
2. Ochiai, T. Gapless surface states originating from accidentally degenerate quadratic band touching. *Phys. Rev. A* **96**, 043842 (2017).
3. Xiao, M., Chen, W.-J., He, W.-Y. & Chan, C. T. Synthetic gauge flux and Weyl points in acoustic systems. *Nat. Phys.* **11**, 920-924 (2015).
4. Bernevig, B. A., Hughes, T. L. & Zhang, S.-C. Quantum spin Hall effect and topological phase transition in HgTe quantum wells. *Science* **314**, 1757 (2006).
5. Liu, C.-X., Qi, X.-L., Zhang, H., Dai, X., Fang, Z. & Zhang, S.-C. Model Hamiltonian for topological insulators. *Phys. Rev. B* **82**, 045122 (2010).
6. Liu, C.-X., Zhang, R.-X. & VanLeeuwen, B. K. Topological nonsymmorphic crystalline insulators. *Phys. Rev. B* **90**, 085304 (2014).
7. He, C. et al. Photonic topological insulator with broken time-reversal symmetry. *Proc. Natl. Acad. Sci. USA* **113**, 4924-4928 (2016).
8. Wu, L.-H. & Hu, X. Scheme for achieving a topological photonic crystal by using dielectric material. *Phys. Rev. Lett.* **114**, 223901 (2015).
9. Alexandradinata, A. & Bernevig, B. A. Berry-phase description of topological crystalline insulators. *Phys. Rev. B* **93**, 205104 (2016).
10. Alexandradinata, A., Holler, J., Wang, C., Cheng, H. & Lu, L. Crystallographic splitting theorem for band representations and fragile topological photonic crystals. Preprint at https://arXiv.org/abs/1908.08541 (2019).
11. Khalaf, E., Benalcazar, W., Hughes, T. & Queiroz, R. Boundary-obstructed topological phases. Preprint at https://arXiv.org/abs/1908.00011 (2019).
12. Blanco de Paz, M. et al. Tutorial: Computing topological invariants in two-dimensional photonic crystals. Preprint at https://arXiv.org/abs/1912.00944 (2019).
13. Wang, H., Guo, G. & Jiang, J.-H. Band topology in classical waves: Wilson-loop approach to topological numbers and fragile topology. *New J. Phys.* **21**, 093029 (2019).
